# Improvement of Aerosol Filtering Performance of PLLA/PAN Composite Fiber with Gradient Structure

**DOI:** 10.3390/nano12224087

**Published:** 2022-11-20

**Authors:** Ping Zhu, Wang Sun, Yunchun Liu

**Affiliations:** School of Instruments and Electronics, North University of China, Taiyuan 030051, China

**Keywords:** the electrode sputters electrospinning technology, gradient structure, PLLA/PAN composite fiber membrane, high humid atmospheric environment, ultrafine aerosol filtration, high production efficiency

## Abstract

Since commercial non-woven air filtering materials have unstable filtering efficiency and poor moisture permeability for the abundant condensed aerosol particles in the highly humid atmospheric environment, the PLLA/PAN composite fiber material with a hydrophobic and hydrophilic gradient structure is designed and prepared by using electrode sputtering electro spinning technology. By characterizing and testing the filtrating effect of SEM, XRD, FTIR, wettability, mechanical property, N_2_ adsorption isotherm, and BET surface area, NaCl aerosol of PLLA fiber, PAN fiber, and PLLA/PAN composite fiber membranes, the study found that the electrode sputtering electrospinning is fine, the fiber mesh is dense, and fiber distribution is uniform when the diameter of the PAN fiber is 140–300 nm, and the PLLA fiber is 700–850 nm. In this case, PLLA/PAN composite fiber materials gather the hydrophobicity of PLLA fiber and the hydrophilicity of PAN fiber; its electrostatic effect is stable, its physical capturing performance is excellent, it can realize the step filtration of gas-solid liquid multiphase flow to avoid the rapid increase of air resistance in a high-humidity environment, and the filtrating efficiency *η* of NaCl aerosol particles with 0.3 μm reaches 99.98%, and the quality factor *Q_F_* 0.0968 Pa^−1^. The manufacturing of PLLA/PAN composite fiber material provides a new method for designing and developing high-performance air filtration materials and a new technical means for the large-scale production of high-performance, high-stability, and low-cost polylactic acid nanofiber composites.

## 1. Introduction

Aerosol, with a variety of nearly spherical shapes, such as liquid droplets, sheets, needles, and other irregular shapes, is the dispersal system formed by solid and liquid particles stably suspended in a gas medium; the particle size of the most penetrating aerosol (MPPS) is between 100 nm and 300 nm [1,2,3]. Aerosols can be divided into hydrophilic and hydrophobic according to their hygroscopicity [4]. Hydrophilic aerosols include inorganic salt aerosols (such as sulfate, nitrate, ammonium salt, and sea salt) and hygroscopic organic aerosols; hydrophobic aerosols consist of black carbon, non-hygroscopic organic aerosols, and dust. Aerosol filtration is mainly carried out by five filtrating mechanisms: filtrating/screening, inertial deposition, diffusion, gravity effect, and electrostatic attraction, which separate and capture solids and liquids in the gas [5,6,7,8]. For aerosol with a particle size of 200~500 nm, when the particles contain covalent compounds, the filtering mechanism is filtrating/screening, inertial deposition, and gravity deposition, and the electrostatic effect plays an auxiliary role; when the aerosol contains ionic compounds, the filtering mechanism is mainly run by electrostatic effect; when the particle diameter is less than 200 nm, Brownian diffusion plays a dominant role, and the filtering mechanism is mostly run by diffusing deposition. The traditional air filter material, generally a single non-woven fiber fabric with a hole diameter from 10 μm to dozens μm, can merely intercept μm level particles; for sub-micron condensed particles, electrostatic trapping is the most effective method [9,10,11], but the number of electrostatic charges of non-woven materials after electret treatment is easy to decay; especially under high temperature and humidity conditions, the decay rate will accelerate [12,13,14]. Therefore, the usual non-woven air filtering materials can no longer meet the requirements of the filtration industry; it is necessary to develop a new filtering material with high efficiency, low resistance, and high stability.

The non-woven air filtering material is a fiber mesh or fiber mat with particular strength and stability formed by various strengthening methods of non-oriented fibers, whose filtering performance depends chiefly on the particular structure of the material itself. The filtering performance of the traditional single-type fiber filtering material is difficult to optimize synchronously and has a single function, gradually failing to meet the application requirements; therefore, researchers have introduced composite technology into the process of high-performance air filtering materials, that is, using different thickness fiber layers to process composite filtering materials with a gradient structure. At present, the preparation methods of non-woven air filtering materials consist of electrospun fiber composite, paper-making, wet forming multilayer composite, melt-blown non-woven fabric composite, and film-covered composite, etc. [15,16], among which electrospun fiber composite and paper-making, wet forming, multilayer composite are the two basic preparation methods for non-woven air filtering materials. Most aerosols under working conditions are a three-phase coexistence of gas, liquid, and solid. The condensed aerosols are supposed to contain both hydrophilic and hydrophobic particles. In this case, the easily functionalized electrospun composite fibers can be effectively captured, but it is difficult to customize the functions of the air-filtering materials formed by paper making. Hence, the advantages of electrospun air filtering materials, such as high specific surface area, adjustable porosity, and easy functionalization, have attracted much attention in air filtration applications. However, traditional electrospinning equipment still uses single/multiple needle devices, whose output can only reach 0.1~1 g/h, resulting in high production costs [17,18]. The reason is that there are many uncontrollable factors in the electrospinning process [19,20], such as (1) Taylor cone indentation inside the spinneret, which increases the jet’s Rayleigh instability, axisymmetric instability, disturbance, and swing instability; (2) Since the electrospun jet lasts for a short time, the polymer macromolecules do not have sufficient time for regular arrangement, and the fiber crystallinity is low, leading to poor mechanical properties of the fiber; (3) The spinning needle tube is easily blocked due to the condensation of the polymer solution.

Many new electrospinning technologies, such as the needle-free electrospinning technology developed by Elmarco corporation [21], the rotating cone as the spinneret proposed by Lu et al. [22], and the use of wire electrodes to replace the spinneret given by Forward, K. M. [23], Indrani, B. et al. [24], have been developed in recent years to solve the problem of large-scale electrospinning production. These methods and technologies all have the common feature of stimulating multiple jets of polymer solution on the surface of a free liquid to improve the production efficiency of nanofibers; commonly referred to as free surface electrospinning, or needle-free electrospinning [25,26]; they are very conducive to the substantial increase of material application performance and the expansion of application fields. However, these studies all aim at the preparation and filtration performance of traditional single-fiber filter materials. The single function of fiber filtering materials makes it challenging to cope with the complex application environment. Therefore, by mixing fibers with different wetting characteristics, the preparation of fiber-mixed filter materials with high efficiency and low resistance can be achieved, which provides theoretical guidance and research basis for exploring new high-performance filtering materials.

In this study, since high humidity, condensed particles are easily formed between inorganic salt aerosol particles, or between inorganic salt particles and other aerosol particles in the atmospheric environment due to the large content of inorganic salt aerosols [27], the electrode sputters electrospinning technology is used to prepare PLLA fiber and PAN fiber successively in a two-step procedure on the substrate with thin micron fiber mesh functioning as the substrate. The composite fiber material has a fluffy stacking structure and a hydrophobic and hydrophilic gradient function, which not only filters aerosols efficiently but also enhances the driving force of air transmission; the filtrating efficiency of the PLLA/PAN fiber membrane has been tested for 300 to 1000 nm NaCl aerosol particles.

## 2. Materials and Methods

### 2.1. Materials

Polylactic acid (PLLA), brand 190, is produced by Zhejiang Haizheng Co., Ltd. (Taizhou, China); Polyacrylonitrile (PAN), powder, is produced by Shanghai Sibaiquan Reagent Co., Ltd. (Shanghai, China); Dichloromethane (DCM), dimethylformamide (DMF) and anhydrous ethanol, all of which are analytically pure, are by Chengdu Kelong Chemical Co., Ltd.(Chengdu, China); the base material is (PE+PPT): 6–18 g/m^2^, filtering effect 70%, moisture resistance ≤90% RH, initial resistance 20–28 Pa, and final resistance ≤80 Pa.

The preparation of PLLA spinning solution: a certain amount of solvent DCM is measured to be put into a conical flask, and then some PLLA is slowly added in the stirred conical flask, lastly, the magnetic stirrer is put in with the bottle cap tightly covered to prevent water vapor from entering the conical flask, which is placed in a water bath pan at 45 °C for stirring. After continuous stirring and dissolution for 1 h, some mass percentage of DMF is added, then continues to be stirred until the solution is clear, and then a specific mass concentration of PLLA spinning solution is obtained. The mass ratio between DCM and DMF is 16:1, and the concentration of PLLA spinning solution is 10%.

The preparation of PAN electrospinning solution: a certain amount of solvent DMF is added into a conical flask, then some PAN is slowly added in, lastly the magnetic stirrer is put in with the bottle cap tightly covered to prevent water vapor from entering the conical flask, which is then placed in a water bath pan for stirring; the water bath temperature is set to 45 °C, and stirred continuously until the solution becomes clear and transparent. The concentration ratio of the PAN spinning solution is 7%.

### 2.2. Design of Composite Fiber

The paper aims to prepare nanofiber materials that can filter aerosols in high-humidity atmospheric environments; the materials can transfer water and capture aerosols efficiently. To achieve efficient filtration, the pores and fiber thickness in the filtering material should match the captured particle size and have a high specific surface area so that the aerosol can be trapped in the nanofiber spatial structure. Hence, filtering materials with a gradient structure are designed according to the following three standards: (1) Fiber materials must be porous and have a high specific surface area; (2) Fiber materials have electrostatic adsorption functions, effectively capturing ionic compounds in aerosols; (3) Composite fiber materials should contain functional hydrophobic fibers to avoid a rapid increase of air resistance in a high humidity environment and contain functional hydrophilic fibers to improve the filtration efficiency of aqueous aerosols. The overall structure of the composite fiber material takes on a hydrophobic and hydrophilic gradient to enhance the driving force of air transmission. The filtration process of a gradient structure composite fiber membrane is shown in the abstract graphic.

According to the above design standards, the PAN fiber is selected as a hydrophilic component for the following reasons [28,29,30,31]: PAN has a high dielectric constant, and more charges are easily generated on the surface of nanofibers under the electrostatic spinning electric field which is conducive to the electrostatic effect of capturing aerosol particles; there are a lot of amide and carboxyl groups in electrospun PAN molecules, which are hydrophilic groups; the N atom on the branch chain of PAN structure has strong electronegativity, which is facilitative to the rapid combination of fibers and positively charged components in aerosols (metal hydroxide and metal oxide particles, such as Fe(OH)_3_, Al(OH)_3_, Fe_2_O_3_, and ZrO_2_, etc.); the diameter range of electrospun PAN fiber is about 200 nm, which easily achieves a fluffy structure, increasing the capturing time of aerosol particles; PAN has excellent solvent resistance, heat resistance, hydrophilicity, mechanical stability, and environmental protection.

Therefore, the introduction of PLLA fiber as a hydrophobic component is for the following reasons [32,33,34]: There are many ester groups in the PLLA molecule, which are hydrophobic; the electrospun PLA fiber has low surface free energy, rough nanoporous structure, and pore size of membrane from 100 nm to 10 mm, which has hydrophobic properties; PLLA is a non-toxic and biodegradable polyester extracted from wheat, potato, and corn; it has excellent biological characteristics and can inhibit the growth of many fungi, yeasts, and bacteria.

### 2.3. Manufacture of Composite Fiber

**Figure 1:**
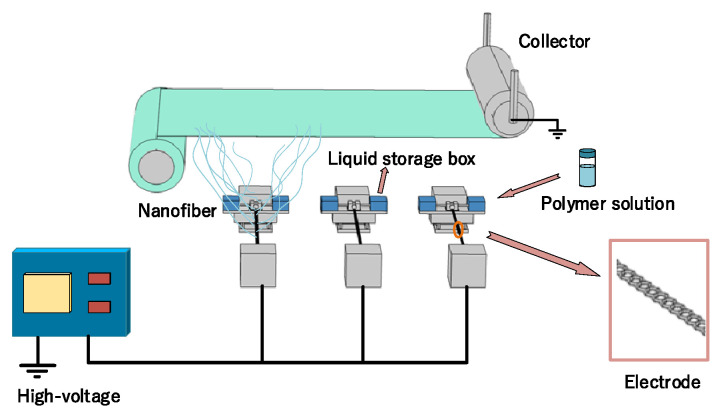
Schematic of electrode sputtering electrospinning device.

Electrospinning adopts an electrode-sputtering electrospinning device produced by Qingdao Junada Company, China, which consists of three parts: jet device, collecting device, and high-voltage power supply. The injection device is comprised of a metal wire electrode group and a liquid supply system, wherein the metal wire electrode group is made up of a plurality of metal wire electrodes placed on the same horizontal plane in parallel, and is connected to the positive pole of the high-voltage power supply; the liquid supply system consists of a fluid supply kettle and a micro brush head placed in the infusion tank; the micro brush head dips the metal wire electrode through continuous movement; the receiving device, connected to the negative pole of the high-voltage power supply is a roller collector, which is assembled on the injection device. The schematic diagram for electrospinning is shown in Figure 1.

The composite fiber material is prepared by a two-step procedure with the spinning environment parameters: temperature, 27~32 °C; humidity, 40 ± 2%. The first step is to electrospin a layer of PLLA fiber. The spinning process parameters of PLLA fiber are as follows: the electrode moving width, 300 mm; the distance between the electrode and the base cloth, 25 cm; the power supply voltage, 45~55 KV. The three electrodes brush liquid simultaneously, the round-trip speed of which is 200 mm/min, and the unwinding rate is 250 mm/min. The second step is to deposit a layer of electrospun PAN fiber on the fiber membrane prepared in the first step. The spinning process parameters of PAN fiber are as follows: the electrode moving width, 300 mm; the distance between the electrode and the substrate, 24 cm; the voltage, 36~42 KV; the brush speed, 150 mm/min; the unwinding rate, 230 mm/min.

The preparation process of the single fiber material should follow a single step of the composite fiber material preparation process. The two steps can also be expanded if additional procedures are required to prepare multilayer composite fiber materials.

### 2.4. Test

The morphologies of the fibers were tested by JSM-6700F (JEOL, Tokyo, Japan) scanning electron microscope (SEM); the fiber diameter distribution was studied by the statistical analysis of 50 fibers randomly selected from SEM using Image-Pro-Plus software. The surface chemical properties of the fiber were analyzed by MAX-2400 X-ray (RIGAKU, Tokyo, Japan) diffractometer (XRD) and EQUINOX-55 (BRUKER, Rheinstetten, Germany) Fourier transform infrared reflection (FTIR). The mechanical properties of the fiber membrane were examined by the XQ-2 (Shanghai New Fiber Instrument Co., Ltd., Shanghai, China) universal mechanical testing machine. For the tensile test, the gauge length and width are 22 × 30 mm, and the tensile speed was 10 mm/min. At five different positions of the sample fiber membrane, each tests the thickness and strength values five times, and then calculates the average values. ASAP 2020 (Micromeritics Instrument Co., Ltd., Norcross, GA, USA) Physical Adsorption Analyzer examined the micro-physical property of the fiber membrane. The filtering effect of the fiber membrane was tested by the UTF003 (UTest Intelligence Tech Co., Ltd., Suzhou, China) filtering-material performance test machine. The fogging dust source was NaCl particles, the mass concentration of particulate matter was 15–30 mg/m^3^, the temperature of the whole test device was 25 ± 5 °C, and the relative humidity was 85–95%.

## 3. Results

### 3.1. Morphology of Fibers

**Figure 2:**
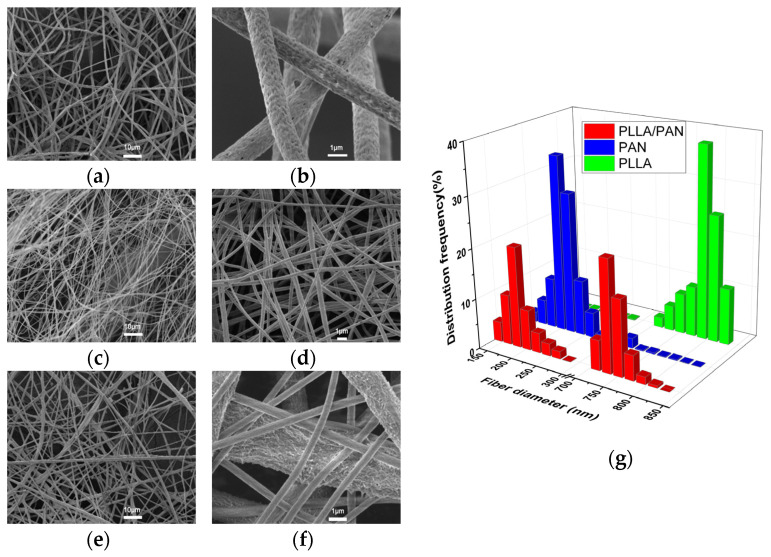
SEM and fiber diameter distribution of the samples, (**a**,**b**) PLLA fiber, (**c**,**d**) PAN fiber, (**e**,**f**) PLLA/PAN composite fiber, (**g**) the fiber diameter distribution.

The SEM images of PLLA fibers, PAN fibers, and PLLA/PAN composite fibers are shown in Figure 2a–f; the average diameter of 50 fibers of the SEM of each sample is calculated; the fiber diameter distribution is shown in Figure 2g; the prepared fibers all present a 3D structure with random orientation, fine spinning, dense fiber mesh, and uniform fiber distribution. As shown in Figure 2a,b, the surface of PLLA fiber is porous, rough, and uniform. The diameter mainly shows a normal distribution at 700–850 nm; the pores of the fiber membrane are large. As shown in Figure 2c,d, PAN fiber is smooth and uniform, and the fiber diameter shows the normal distribution is mainly at 140–300 nm. When the fiber diameter decreases, the distance between fibers decreases, and the pores formed by fiber lapping also decrease, which dramatically impacts the slip effect, which also helps reduce the air resistance of the fiber membrane. As shown in Figure 2e,f, the diameter of composite PLLA/PAN composite fibers forms a bimodal distribution. PLLA fibers are the 3D structural skeleton of the membrane, and PAN fibers penetrate the 3D structure of the membrane with uniform distribution, indicating that the fiber membrane has achieved a structural gradient. The fiber membrane morphology can be optimized by adjusting the spinning time ratio of PLLA/PAN composite fibers.

In the PLLA/PAN composite fiber structure, PLLA fiber provides a stable structural cavity for the composite material and sufficient filtering channels for the larger formed pore structure, ensuring structural stability and mitigating filtering resistance during the filtering process; meanwhile, it can effectively protect the system of PAN fiber and guarantee that PAN fiber maintains stable filtrating performance. In addition, as a reinforcing phase for the interception of small particles, PAN fiber can interfere with the movement track of aerosols in the composite material, increasing the chance of collision between particles and fibers, thus significantly improving the interception and capture of small particles by fiber materials. The above data prove that the composite fiber membrane has achieved a structural gradient, and its filtering behavior is a “Surface + Deep” mode, which is conducive to strengthening the screening effect on particles and ensuring the high efficiency and low resistance filtering performance of composite fiber on aerosols.

### 3.2. Wettability of Fibers

**Figure 3:**
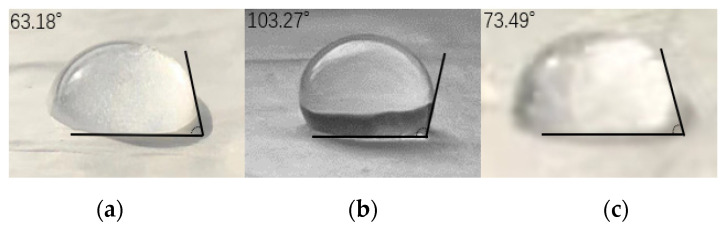
Contact angle diagram of samples, (**a**) PAN, (**b**) PLLA, (**c**) PAN + PLLA.

The surface hydrophobicity of materials can be evaluated by the contact angle; the contact angle diagram of PLLA fibers, PAN fibers, and PLLA/PAN composite fibers is shown in Figure 3.

The contact angle of the PLLA fiber membrane is 103.27°, greater than 90°, which means the electrospun PLLA fiber membrane is hydrophobic on the surface and belongs to hydrophobic material; since PLLA contains many ester bonds, its hydrophilicity is relatively poor, and the surface of the fiber is porous and rough, the PLLA fiber membrane has good hydrophobicity. The contact angle of the PAN fiber membrane is 63.18°, less than 90°, which indicates that the electrospun PAN fiber membrane is hydrophilic on the surface and belongs to hydrophilic material. The contact angle of the PLLA/PAN composite fiber membrane is 73.49°, which is hydrophilic.

The reason that the resistance of PAN fiber increases rapidly under high humidity is because of the hydrophilic property of PAN fiber. When water vapor or tiny water droplets in the air encounter hydrophilic PAN fibers, water will be adsorbed on the fibers and form capillary water between the fibers, blocking the pores of the fibers, causing the air to pass through the fibers unevenly, leading to a rapid increase in resistance. However, in the 3D structure of composite fibers, hydrophilic PAN fibers form a cross structure between hydrophobic PLLA fibers; capillary water formed between PAN fibers can easily flow out along PLLA fibers, reducing the risk of fiber pore plugging.

### 3.3. Chemical Properties and Mechanical Property

**Figure 4:**
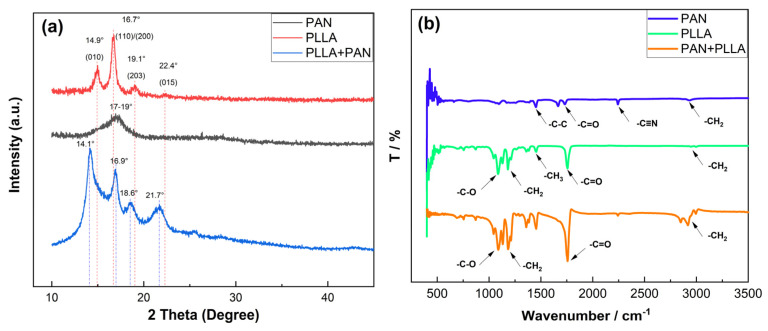
(**a**) XRD patterns of fibrous membranes, (**b**) FTIR patterns of fibrous membranes.

The mechanical properties of electrospun polymer films depend chiefly on crystal structure and morphology. A weak mechanical property is one of the drawbacks of electrospun metastable crystalline polymer film, which can be solved by improving its crystallization degree. The crystallization state of membranes can be analyzed qualitatively by the XRD patterns of samples in Figure 4a.

The crystallization of PLLA electrospun will generate two crystal forms: relatively perfect and stable crystal forms α, and metastable crystal form with imperfect crystallization α’. Here, The crystal peaks of PLLA fiber appear at 2θ = 14.9°, 16.7°, 19.1°, and 22.4°, corresponding to (010), (110)/(200), (203), and (015) crystal plane indexes, respectively. The maximum crystallization peak is at 2θ = 16.7°, corresponding to the (110)/(200) plane. The firm diffraction peaks between 2θ = 14.9°, and 22.4° degrees are associated with the semicrystalline PLLA diffractions. The firm diffraction peaks between 2θ = 14.9° and 22.4° degrees are associated with the semicrystalline PLLA diffractions [35,36,37].

The most substantial reflection, relatively sharp, is observed in PAN fiber between 2θ = 17° and 19°, where the crystallization peak presents a broad steamed bread peak; it indicates that the electrospun PAN fiber is mainly composed of amorphous components, showing that the crystallization of PAN is delayed during electrospinning due to rapid solidification at high elongation [38].

Finally, the crystallization peaks of PLLA/PAN composite fiber appear at 2θ = 14.1°, 16.9°, 18.6°, and 21.7°, and the positions of the crystallization peaks shift and become sharper. The maximum crystallization peak is at 14.1°, suggesting that the crystal form is a stable α crystal form; the other main peak appears at 16.9°; the crystallization peak becomes sharper, and the crystallization strength increases. The crystallization peak at 2θ = 22.4° shifts to 21.7°. These cases suggest that when PLLA fiber enters the high-voltage electrostatic field for the second time, it is equivalently a process of annealing, which can promote the crystallization of PLLA, making a stable α crystal structure of the composite fiber more perfect.

The chemical structure evolution of PAN fiber, PLLA fiber, and PAN/PLLA composite fiber is studied by FTIR spectrum, as shown in Figure 4b. In the infrared spectrum of PAN fiber, the absorption peak intensities are near 2940 cm^−1^, 2248 cm^−1^, 1715 cm^−1^, 1460~1440 cm^−1^, 1270~1220 cm^−1^, representing the stretching vibration absorption peak of methylene (–CH_2_), the stretching vibration absorption peak of cyano (–C≡N), the stretching vibration absorption peak of carbonyl (–C=O), and the variable angle vibration of different forms of methylene (–C–H), respectively. PAN dissolved from DMF solution often has a peak at about 1715 cm^-1^, which is attributed to the vibration of the carbonyl (–C=O) bonds formed in the hydrolyzed PAN fibers and the stretching vibration of the carbonyl bonds in residual DMF solvent [29].

In the infrared spectrum of PLLA fiber, the intensities of absorption peaks, near 2900~3000 cm^−1^, 2876 cm^−1^, 1755 cm^−1^, 1445 cm^−1^, 1355 cm^−1^, 1130 cm^−1^, and 1090 cm^−1^, correspond to the stretching vibration absorption peak of methylene (–CH), the weak absorption peak of methylene (–CH_2_–), the stretching absorption peak of carbonyl (–C=O), the bending vibration absorption peak of the methyl group (–CH_3_), and the vibration absorption peak of C–C bond, the bending vibration absorption peak of methylene methyl (=CH_2_), and the stretching vibration absorption peak of ketone group (–C–O), respectively [39].

In the infrared spectrum of the PLLA/PAN composite fiber, the position of the characteristic absorption peak of the hybrid fiber remains unchanged within the corresponding wave number, without red or blue shift, indicating that the extreme and structure have not changed fundamentally during the secondary electrospinning process. However, the intensity of the infrared characteristic absorption peak has increased. For instance, the weak absorption peak of C–H stretching vibration at 2900~3000 cm^−1^ and 2876 cm^−1^ and the soft absorption peak of methylene (–CH_2_) are enhanced; the tensile absorption peak of cyano (–C≡N) at 2248 cm^−1^ and the characteristic absorption peak of carbonyl (–C=O) at 1755 cm^−1^ and 1715 cm^−1^ are superposed together to increase the amplitude, strengthening the hydrophilicity of PAN fibers; there is a prominent absorption peak near 2900–3000 cm^−1^, the absorption band moves to 3292~3358 cm^−1^, and the height also becomes broader and more robust. Therefore, because methylene and carbonyl groups also exist in the PAN structural formula, some characteristic peaks overlap with PLLA fiber and the peak value increases, but no new bonds are formed, suggesting that PAN fiber and PLLA fiber are a pure physical mixture, relatively independent, and do not generate biochemical bond binding.

The tensile strain-stress tables of the electrospun nano-fibrous membrane are summarized in Table S1. In the process of stretching, the mechanical properties of two kinds of fibers are superior to that of a single fiber membrane due to the interaction of the self-organization of both types of fibers at multiple scales. The strains of PLLA fiber, PAN fiber, and PLLA/PAN composite fiber membranes are 20.52%, 56.17%, and 65.34%, respectively; the stresses are 6.29 MPa, 9.34 MPa, and 11.76 MPa. The test result is based on the reasons that the electrospun fibers are not cross-linked and fixed but interlaced, leading to the weak mechanical properties of a single fiber membrane. In the PLLA/PAN composite fiber membrane structure, the PLLA fiber acts as a solid 3D frame, and the thin PAN fiber fills the 3D network structure, causing wrinkles and dense structures. In addition, PLLA fiber achieves a secondary crystallization during the preparation of PAN fiber; as a result, its mechanical properties are improved.

### 3.4. N_2_ Sorption Isotherm and BET Surface Area Analysis

**Figure 5:**
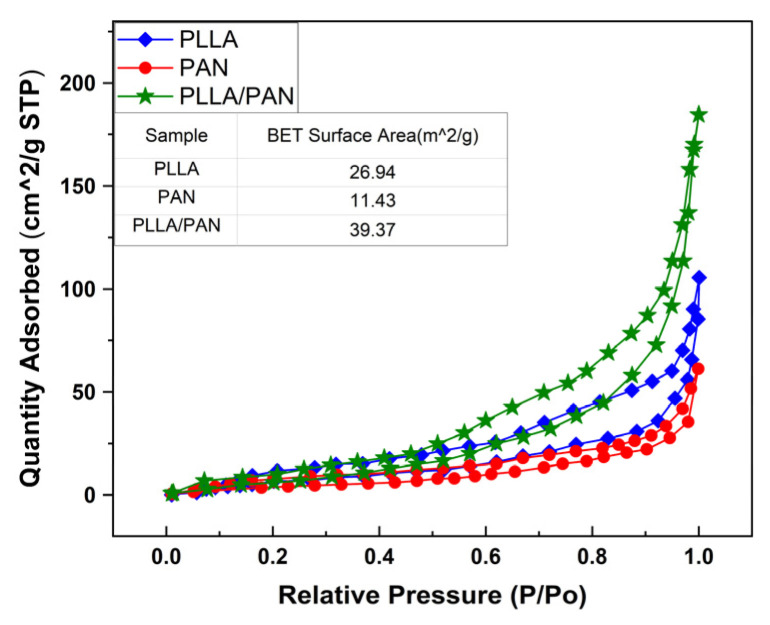
N_2_ adsorption−desorption isotherms graphs of fibrous membranes.

N_2_ adsorption analysis is carried out on three kinds of fiber membranes as shown in Figure 5. This physical characterization method can accurately analyze the microporous and mesoporous structures with pore diameters less than 50 nm. It can be seen from Figure 5 that when P/P_0_ is small, the curve increases slowly; in the middle section of the curve, there is an adsorption hysteresis loop, which corresponds to the capillary condensation of the porous adsorption system, suggesting that the pores in these membranes are filled and emptied. When P/P_0_ is large, the latter part of the curve bulges once more, implying a sudden increase in adsorption capacity. These cases reflect that the three fiber membranes are nonporous, or a single multilayer reversible adsorption process on the microporous adsorbent. Under the IUPAC classification, the desorption isotherms of the three fiber membranes should be recognized as typical type IV isotherm materials. It can be seen from the table shown in Figure 5 that the BET surface areas of PLLA fiber, PAN fiber, and PLLA/PAN composite fiber membranes are 26.94 m^2^/g, 11.43 m^2^/g, and 40.37 m^2^/g, respectively, among which the BET surface area of PLLA/PAN fiber membranes is relatively high, and the physical adsorption capacity is strong.

### 3.5. Aerosol Filtration Analysis

**Figure 6:**
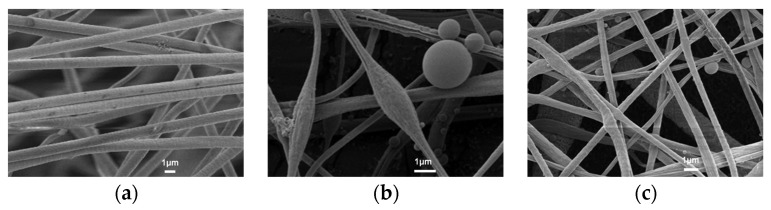
SEM images of samples after filter test with NaCl aerosol, (**a**) PLLA fiber, (**b**) PAN fiber, (**c**) PLLA/PAN composite fiber.

The particle size of approximately 0.3 μm, 0.5 μm, and 1 μm is used to test the filtrating effect of m NaCl aerosol particles on the three materials, as summarized in Table 1. To visually represent the capturing capacity of the three particle materials, the polluted film containing many aerosol particles is cleaned and regenerated through mechanical vibration and air backwashing; the SEM image is shown in Figure 6.

Filter tests are conducted on PLLA fiber, PAN fiber, and PLLA/PAN composite fiber membrane samples. The detailed data of average filter efficiency and pressure drop are calculated, as summarized in Table 1. When the aerosol particle size is 1 μm, the filtrating efficiency is almost 100%, seen in Table 1. In addition, when the size of aerosol particles is ≤0.3 μm with a pressure drop at 90~130 Pa, the filtrating effect is excellent. Previous reports show that the elements of high efficiency and high-quality [*Q_F_* = −ln (1 − *η*)/ΔP, among which *η* and ΔP refer to filtration efficiency and pressure drop] is crucial for better filtrating performance. According to 0.3 μm aerosol particle filtrating efficiency, the *Q_F_* of three samples is calculated, as summarized in Table 1. Based on the previous analysis of fiber diameter and BET surface area, PLLA/PAN composite fiber membrane has a remarkable filtering effect with a filtering efficiency of 99.98% and a quality factor of 0.0968 Pa^−1^. The *Q_F_* and filtration efficiencies of the result are higher than the *Q_F_* (0.034 ± 0.002 Pa^−1^) and the filtration efficiencies (95.97 ± 0.62%) of the membrane of PAN with cellulose nanocrystals reported by by Rachel Passos de Oliveira Santos et al. in February 2022 [40].

In this case, the gradient structure of this composite fiber membrane ensures high surface adsorption and fluffy stacking structure, increasing the travel time of aerosols between the 3D network gaps of the fiber, making the deposition behavior more adequate, and improving the filtering efficiency of aerosols; besides, at the interface between various coarse and fine fibers in the gradient structure, the porosity and permeability change continuously and rapidly, which is conducive to the formation of air pressure difference, making the convection flow in the fiber no longer symmetrical, promoting the increase of the velocity gradient of air convection, and then producing the inertial stress slip effect, which is helpful to reduce the air resistance of the fiber membrane. Therefore, PLLA/PAN composite fiber membrane shows lower air resistance.

After the NaCl aerosol filtrating test, three samples were tested by SEM to compare the deposition behavior of NaCl aerosol particles in the three materials, as shown in Figure 6. In Figure 6, no particles are deposited on the surface of PLLA fiber. More particles are deposited on the surface of PAN fiber and PLLA/PAN composite fiber, among which the aerosol particles deposited on PAN fiber are larger. It fully confirms that the PLLA fiber, with good hydrophobicity, does not electrostatically absorb NaCl aerosol particles; rather, the aerosol particles are deposited on the fiber by filtrating/screening, inertial deposition, diffusion, gravity effect, etc., and there is no residue on the fiber after cleaning; then, the PAN fiber membrane has excellent electrostatic adsorption capacity. In addition to physical deposition, aerosol particles can be adsorbed on the fiber by electrostatic effect, and there are many residues on the fiber after cleaning. Through the combination of physical filtration and chemical filtrating mechanisms, the PLLA/PAN composite fiber material with a hydrophobic and hydrophilic gradient structure can quickly transfer water, making the step filtration of the gas-solid-liquid multiphase flow occur, and avoiding the rapid increase of air resistance in high humidity environment.

## 4. Conclusions

In this work, PLLA/PAN composite fiber with a hydrophobic and hydrophilic gradient structure was designed and prepared by electrospinning with electrode sputtering. Through characterizing and performing tests of PLLA fiber, PAN fiber, PLLA/PAN composite fiber membranes, and NaCl aerosol filtrating tests, the conclusions can be drawn as follows: The electrode sputter electrospinning is fine, the fiber mesh is dense, fiber distribution is uniform, and spinning efficiency is high. PLLA/PAN composite fiber material has the hydrophobicity of PLLA fiber and the hydrophilicity of PAN fiber; its electrostatic effect is stable, its hydrophobicity good, its physical capture performance is excellent, the filtrating effect of NaCl aerosol the best, and the production efficiency is high. The filtrating efficiency *η* of NaCl aerosol particles with 0.3 μm reaches 99.98%, and the quality factor *Q_F_* is 0.0968 Pa^−1^ at the relative humidity of 85–95%. By enhancing the mechanical properties of the filter material and improving the functionality of the membrane with a gradient structure, the working environment can be expanded, being widely used in the life and production of air filtration, including automobile exhaust, room cleaning, ultra-low penetration air filters, and respiratory protection equipments.

## Figures and Tables

**Table 1:** Filtering effect of samples.

Sample	Filtration Efficiency (*η*/%)			Thickness/μm	∆P/Pa	*Q_F_*
	0.3 μm	0.5 μm	1 μm			
**PAN**	99.626	99.905	99.991	48 ± 2	127 ± 8	0.0441
**PLLA**	99.534	99.722	99.916	41 ± 2	107 ± 6	0.0502
**PLLA/PAN**	99.985	99.997	99.998	44± 2	91 ± 3	0.0968

## Supplementary Materials

The following supporting information can be downloaded at: https://www.mdpi.com/article/10.3390/nano12224087/s1. Table S1: Stress−strain curves of fibrous membrane.
