# Supplementary material for: Improvement of Aerosol Filtering Performance of PLLA/PAN Composite Fiber with Gradient Structure
Source: Nanomaterials (Basel). 2022 Nov 20;12(22):4087. doi: 10.3390/nano12224087 (PMC9697973; doi:10.3390/nano12224087)
Supplement: Supplementary file 1 [file nanomaterials-12-04087-s001.zip › nanomaterials-2047875-supplementary.pdf]

Supplementary Materials

Improvement of Aerosol Filtering Performance of PLLA/PAN Composite Fiber with Gradient Structure

Ping Zhu *, Wang Sun and Yunchun Liu

School of Instruments and Electronics, North University of China, Taiyuan 030051, China

* Correspondence: h.zhuping@163.com; Tel.: +86-03513922540

Table S1. Stress–strain curves of fibrous membrane

	PLLA	PAN	PLLA/PAN
Strain (%)	20.52	56.17	65.34
Stress (MPa)	6.29	9.34	11.76
